# Gender differences in ADHD and impulsivity among alcohol or alcohol- and cocaine-dependent patients

**DOI:** 10.3389/fpsyt.2025.1446970

**Published:** 2025-02-27

**Authors:** Carlos Roncero, Diego Remón-Gallo, LLanyra García-Ullán, Begoña Vicente-Hernández, Barbara Buch-Vicente, Raul Felipe Palma-Álvarez, Lara Grau-López, Kristofer Ramon González-Bolaños, Ana Álvarez-Navares, Jésus Pérez, Lourdes Aguilar

**Affiliations:** ^1^ Department of Health Sciences, Miguel de Cervantes European University, Valladolid, Spain; ^2^ Psychiatry Unit, School of Medicine, University of Salamanca, Salamanca, Spain; ^3^ Institute Carlos III, Network of Research In Primary care of Addictions (RIAPAD), Madrid, Spain; ^4^ Institute of Biomedicine of Salamanca (IBSAL), Salamanca, Spain; ^5^ Psychiatry Service, University Health Care Complex of Salamanca, Salamaca, Spain; ^6^ Department of Basic Psychology, Psychobiology, and Methodology of Behavioral Sciences, Faculty of Psychology, University of Salamanca, Salamanca, Spain; ^7^ Department of Psychiatry and Legal Medicine, Universitat Autonoma de Barcelona, Bellaterra, Spain; ^8^ Group of Psychiatry, Mental Health and Addiction, Vall d’Hebron Institut de Recerca (VHIR), Barcelona, Spain; ^9^ Biomedical Network Research Centre on Mental Health (CIBERSAM), Barcelona, Spain; ^10^ Department of Psychiatry, Hospital Universitari Vall d'Hebron, Barcelona, Spain

**Keywords:** gender differences, addiction, alcohol, cocaine, impulsivity, ADHD

## Abstract

**Background:**

Impulsivity plays a fundamental role in the realm of addiction as is considered a risk factor for addiction. Moreover, it influences the age of onset, severity, and therapeutic management of addictions. The aim of this study was to explore measures of impulsivity in a cohort of male and female diagnosed with Alcohol Use Disorder (AUD) and contrast these findings with those from a group with Alcohol and Cocaine Use Disorder (ACUD).

**Methodology:**

A total of 204 patients (153 men and 51 women) underwent evaluation using Adult ADHD Self-Report Scale (ASRS), Barrat Impulsiveness Scale (BIS-11), Zuckerman-Kuhlman Personality Questionnaire (ZKPQ), Visual Analogue Scale (VAS), Beck Depression Inventory (BDI) and State-Trait Anxiety Inventory (STAI).

**Results:**

A total of 24.6% of the sample (21.9% AUD group and 32.2% ACUD group) screened positive for ADHD. Differences were observed in Total Impulsivity (T(199) =-2.587, p=.010), with the mean score being higher in the ACUD group. Gender differences were noted with ADHD exhibiting a significant explanatory power for impulsivity (greater than 37%) in women compared to men, where its relevance is minimal. Among women, an inverse relationship was found between impulsivity and activity and sociability, in contrast to men, where the inverse relationship was with intolerance to isolation. Both men and women showed associations between ADHD and elevated levels of anxiety and depression. Study limitations and practical implications are discussed.

**Conclusions:**

Although this is an observational study and should be develop a longitudinal study, we detected that the presence of ADHD in addicted women significantly influences impulsivity and should be systematically assessed due to the differences in the clinical approach.

## Introduction

Impulsivity is a personality trait defined as a predisposition toward unplanned reactions without regard to the negative consequences of these reactions to the individual or others. Several studies have attempted to explain impulsivity and its association with several psychiatric disorders, including addictive behavior (1–3). A systematic meta-review even points out that impulsivity is an essential part of substance and behavior addictions, rather than a mere consequence of them (4).

Drug use is associated with cognitive and neurological deficiencies, many of which persist after their use is interrupted (5). One aspect of cognition affected by the addiction is the decision-taking process, so that small short-term gains are selected over larger long-term gains. This “impulsive choice” may be associated with an inability to adequately assess the consequences of actions (6). In addition, impulsivity is associated with attention, memory and approach biases in patients with substance use disorders (SUDs) (7). On the other hand, there are studies that have shown that a prolonged use of cocaine increases impulsive behavior (8).

Substance misuse shows differences regarding gender (9, 10). Several researchers have observed that women could be more vulnerable to addiction, with data showing that they increase rapidly the amount they use, as has been described in the case of alcohol, most illegal substances and gambling (11, 12). Gender differences have also been reported among alcohol-dependent patients, including behaviors linked to impulsivity such as suicidal attempts (9).

Other authors have pointed out that men are generally more impulsive than women and have more associated psychiatric disorders (13, 14). Women are more sensitive to punishment, which makes them present less risky behavior (15), while men take greater risks and seek out new sensations (16).

Alcohol consumption has been associated with high levels of impulsivity (17, 18). In this regard, alcohol consumption expectation and impulsivity were the best predictive factors for substance use disorders in both men and women (19). Patients with the greatest impulsivity reported higher levels of intention to drink and alcohol consumption (20). However, some studies show that women with alcohol use disorder present greater impulsivity than men (21). This could be explained by the fact that, compared to men, women are more susceptible to the effects of chronic alcohol consumption, with alterations in the frontal lobe and greater impulsivity after prolonged use (22). The impulsivity levels may also vary based on the substances that have been consumed (23, 24).

Regarding impulsivity in cocaine users, men showed higher scores in the search for emotions than women (25). The association between cocaine consumption and impulsivity is bidirectional. Impulsivity may be a risk factor for cocaine consumption and, in turn, cocaine dependence increases impulsivity (3). In addition, a study has reported that more severe patients with cocaine-induced psychosis show higher levels of impulsivity and a higher prevalence of Attention Deficit Hyperactivity Disorder (ADHD) (3).

ADHD is a disorder that starts in childhood and is associated with multiple disorders (26), including drug use (27) or drug addiction (28). One of the main symptoms of ADHD is impulsivity, which has been associated with an increased use of alcohol and alcohol and substance use disorders (29). Women with ADHD are thought to be less vulnerable than men, but their involvement in drug use is the same (30). Alcohol use itself increases impulsivity, creating a vicious cycle where ADHD-related impulsivity leads to alcohol consumption, which leads to more impulsivity and to binge drinking and loss of control. Furthermore, adolescents with ADHD, especially those who are not being treated, are more vulnerable to the reinforcing effects of alcohol (17, 18).

The concurrence of ADHD and substance use disorder (SUD) is associated with higher levels of impulsivity (3), and an early appearance of the addiction (31), although the direction of causality, the underlying mechanisms, the clinical implications of the strong association between ADHD and SUD and the influence of gender are still unclear.

This article analyzes the influence of gender, impulsivity and ADHD on patients with Alcohol Use Disorder (AUD) and Cocaine Use Disorder (CUD). The hypothesis is that ADHD may have an influence on higher levels of impulsivity for the consumption of cocaine and alcohol, with greater effect on women.

## Materials and methods

The study was conducted in the Outpatient Alcohol and cocaine Clinic of the Psychiatry Service of the Salamanca´s University Hospital in Salamanca. The sample included 204 patients (153 men and 51 women) seeking treatment for Alcohol Use Disorder (AUD) (n=150) and for AUD and Cocaine Use Disorder (CUD) (n=54) according DSM-5 criteria. The proportion of men and women reflected the reality of the outpatient treatment unit where this study took place. The exclusion criterion for patients with severe mental illness (such as psychosis or bipolar disorder) was considered because these patients tend to present more severe psychopathology and specific symptoms of the other mental disorders and a severe organic comorbidity, as well as more medication, which in many cases prevents them from successfully completing the self-administered assessment.

### Participants

Data were collected between January 2020 and January 2022. Inclusion criteria were the following; patients must be 18 years or older, AUD with or without CUD, provide signed informed consent, finish the test evaluation process. Exclusion criteria were having previous diagnoses of psychosis or bipolar disease, not having a fluent Spanish expression or comprehension. The study protocol was approved by the Hospital committee (PI 2020 10 603). Patients did not receive monetary compensation for their participation in the study.

### Methodology

The assessment was conducted in a single session, during which participants completed a battery of self-report measures. Throughout the evaluation process, participants were accompanied by a trained psychologist to address any questions that might arise while completing the tests. The average time to complete the full battery was approximately one hour. However, there is some variability in participants’ completion times (between 1-2 hours). Incomplete tests (>10% of items missing) were excluded from the analysis. Therefore, the degrees of freedom vary between comparisons. For up to 10% missing items, intermediate scores were used. The self-report measures used in this study are as follows.

### Adult ADHD Self-Report Scale (ASRS)

The 6-question self-report screening questionnaire ASRS-v1.1 (Adult ADHD Self-Report Scale, available at: http://www.hcp.med.harvard.edu/ncs/asrs.php) was developed jointly by the WHO and doctors Kessler, Adler and Spencer (32). ASRS-v1.1 is a subgroup of the symptom’s checklist of the 18-question WHO questionnaire. It is based on the diagnostic criteria of the Diagnostic and Statistical Manual of Mental Disorders (DSM-IV-TR) of the American Psychiatric Association (33). In Spanish a validated version has been published. It concluded that the ASRS-v.1.1 is an effective tool for the initial screening and that its items measure a nonspecific dimension of compulsiveness/impulsiveness, when it uses the 4-point cut-off. The values obtained for sensitivity (87.5%) and specificity (68.8) indicate that it is a useful test and that it achieves its objective as a screening tool in a drug-dependent population. It should be mentioned that considering a cut-off equal to or greater than 3 results in greater sensitivity (93.8%), which could be clinically relevant when identifying patients with ADHD under treatment with SUD (34). The variable obtained from the test is the total score, which can range from 0 to 24. The cutoff score to assess a potential ADHD diagnosis is 13 points.

### Barrat Impulsiveness Scale (BIS-11)

The Spanish version (35, 36) was completed by all patients. The BIS-11 is a measure of “trait impulsivity”. This self-administered questionnaire provides a total score and three subscales’ scores. These four scores were used as dependent variables: cognitive impulsivity (tendency to make quick decisions); motor impulsivity (propensity to act solely for the stimulus without thinking of the consequences) and unplanned impulsivity (high interest for the present that the future). All items are measured on a 4-point scale (0 = Rarely/Never; 1= Occasionally; 3= Often; 4= Almost Always/Always). The items are summed and the higher the BIS-11 total score, the higher the impulsiveness level is 12 out of the 30 items are reverse order to avoid response bias. BIS-11 shows high reliability (Cronbach’s alpha = .81) in it´s Spanish adaptation.

### Zuckerman-Kuhlman Personality Questionnaire (ZKPQ)

The Spanish version (37) of this questionnaire consists of five scales. (1) Neuroticism–Anxiety (N–Anx, 19 items); (2) Activity (Act, 17 items); (3) Sociability (Sy, 17 items); (4) Impulsive Sensation- Seeking (ImpSS, 19 items); and (5) Aggression–Hostility (Agg–Host, 17 items). The ZKPQ also includes an Infrequency scale (Infreq, 10 items). It is answered with True or False, so the maximum score for each scale ranges from 0 to the total number of items in the scale. There are no cutoff points, as it is a test of non-pathological personality traits based on the author’s personality theory. Impulsive Sensation-Seeking (ImpSS, 19 items) items involve a lack of planning and the tendency to act without thinking and the seeking of excitement, novel experiences and willingness to take risks for these types of experiences. The ImpSS scale can be separated into two facets: impulsivity (Imp, 8 items) and sensation seeking (SS, 11 items), providing a more conceptually and empirically refined discrimination of drug-dependent patients. The subdivision also applies to Activity scale (General Activity and Working Effort subscales) and Sociability scale (Party and Friends, and Isolation Intolerance subscales).

### Visual Analogue Scale (VAS)

A VAS (Visual Analogue Scale) was used at the beginning of treatment to evaluate patients’ craving level during the last month. The scale uses a horizontal line without markings where patients must place a mark indicating the intensity of their craving. A mark at the beginning of the line represents the complete absence of craving, while a mark at the end represents the maximum craving. This line measures 10 cm and uses a scoring scale from 0 to 10.

### State-Trait Anxiety Inventory (STAI)

State–Trait Anxiety Inventory (STAI): Self-administered questionnaire that measures the current anxiety symptoms of the patient. Spanish adaptation by Gualberto Buela-Casal, Alejandro Guillén-Riquelme, and Nicolás Seisdedos Cubero was used (38). This self-report consists of 40 questions designed to evaluate the two scales it comprises (20 questions each scale). Each item is answered using a Likert scale from 0 to 3, so the maximum score for each test is 60. The first scale is STAI-State, which refers to the sensations of anxiety experienced by the subject while completing the test. STAI-Feature aims to measure daily anxiety in subjects´ life.

### Short form of the Beck depression inventory I and Beck depression inventory II

The assessment protocol initially included the first short form of the Beck Depression Inventory (39). This version includes 13 questions and shows a score between 0 and 39 points. The cutoff points refer to the different levels of depression: absent or minimal depression (0-4), mild depression (8-15), and severe depression (>15). However, it was necessary to change to the Spanish adaptation of the current version; the Beck Depression Inventory II (40). This version includes 21 questions, with a final score between 0 to 63. Although cutoff values change, the levels of depression are the same as in the previous version; absent or minimal (0-13); mild (14-18), moderate (19-27), and severe (28-63). In the analysis of depression as a variable, a value was assigned to each level: absent-minimal (0), mild (1), moderate (2) and severe (3). Therefore, the analysis in this study was conducted by classifying patients into groups with a score of 0-3.

### Data analysis

A descriptive analysis of all sample variables was conducted, including measures of mean, median, standard deviation, interquartile range, and normality assessment through skewness and kurtosis. Subsequently, an analysis with two independent samples was performed (Student´s t-test) to determine whether the variables regarding to compulsiveness/impulsiveness (BIS-11) and ADHD (ASRS) were associated with gender. Levene test was used to check homoscedasticity criteria, using a significance of.05. Although they are independent tests, some instruments break down their scores into subscales. In these cases it was applied the False Discovery Rate (FDR), especially used as a correction for Type I Error in cases where multiple analyses are conducted to control the proportion of false positives among the significant results. These are the cases of BIS-11, ZKPQ Activity, Sociability and Impulsivity, and STAI State and Feature. The analysis of these variables was repeated using the type of drug consumed as the Independent Variable.

Second part of the study involves analyzing the percentage of participants who scored above the cutoff point on the ASRS test. For this analysis, Chi-Square tests were conducted for Gender and Type of Substance. In both tests, the dependent variable was the frequency of participants scoring above the cutoff point (13 or higher), and the independent variable was the substance use group (alcohol vs. alcohol and cocaine). The third part on the analysis consisted on exploring the relationship between ADHD (measured with ASRS) and impulsiveness (BIS-11). It was also explored the relationship between ADHD and variables measured with ZKPQ. For this purpose, Pearson correlation was applied. FDR was also calculated in those correlations p, in order to statistically control multiple analysis in the same test.

All analyses were conducted in SPSS v. 28.0.1.1, except for FDR, which was calculated using the Benjamini-Hochberg formula.

## Results

Independent sample T test reported (see **Table 1**) significant differences in Neuroticism by male and female, t(200)=-2,382, p=,018, 95% C.I. (-3,383/-,319). Female are drawing on an average higher neuroticism (M=11,75, SD=5,023) as compared to male (M=9,89, SD=2,720). Working Effort as subscale from Activity reported differences between male and females, but when FDR correction was applied, differences show to be not significant. The same occurred with Search of Sensation as subscale from Impulsivity. Craving for cocaine reported significant differences by male and female, t(165,24)=2,292, p=,023, 95% C.I. (,891/1,976). In this case women show on average less craving for cocaine (M=,320, SD=1,316) than men (M=,963, SD=2,573). STAI-Feature showed, on average, higher levels on women (M=32,10, SD=13,02) than men (M=27,08, SD=12,82).

**Table 1:** Mean scores between men and women for the main tests applied.

	Mean (S.d)			p	FDR
	Total (n=204)	Men (n=153)	Women (n=51)		
**AGE**	48,00 (11,11)	47,74(11,09)	48,79 (11,24)	,553	–
**TOTAL BIS-11**	69,59 (11,18)	69,57 (11,19)	69,38 (11,21)	,919	,919
Cognitive Impulsivity	20,63 (3,82)	20,69 (3,60)	20,34 (4,41)	,579	1,152
Motor Impulsivity	22,06 (4,79)	21,96 (4,66)	22,10 (4,87)	,856	1,141
No planeada Impulsivity	27,22 (4,93)	27,42 (4,62)	26,76 (5,74)	,413	1,653
**ASRS**	10,44 (5,72)	10,34 (5,97)	10,49 (4,58)	,869	–
**ZKPQ NEUROTICISM**	10,42 (4,87)	**9,89 (4,72)**	**11,75 (5,02)**	,018*	**-**
**ZKPQ ACTIVITY**	8,77 (3,51)	8,88 (3,58)	8,41 (3,32)	,411	,616
General Activity	5,13 (2,20)	5,09 (2,19)	5,22 (2,28)	,718	,718
Working Effort	3,65 (1,83)	**3,8 (1,87)**	**3,20 (1,65)**	,041*	,123
**ZKPQ SOCIABILITY**	6,00 (3,56)	5,97 (3,73)	5,98 (3,08)	,991	,991
Party and Friends	2,71 (1,96)	2,77 (2,04)	2,43 (1,65)	,287	,861
Isolation Intolerance	3,30 (2,24)	3,21 (2,24)	3,55 (2,17)	,342	,342
**ZKPQ IMPULSIVITY**	8,70 (4,07)	8,97 (4,16)	8,05 (3,33)	,096	,144
Impulsivity	3,73 (1,91)	3,71 (1,97)	3,78 (1,66)	,784	,784
Search of sensations	4,98 (3,01)	**5,26 (3,00)**	**4,16 (2,89)**	,023*	,069
**ZKPQ AGGRESSIVESNESS**	7,53 (3,24)	7,48 (3,14)	7,57 (3,52)	,871	–
**ZKPQ INACCURACY**	2,25 (1,72)	2,32 (1,72)	2,04 (1,62)	,314	–
**Analogue Evaluation Alcohol**	2,86 (3,32)	2,960 (3,31)	2,674 (3,39)	,600	–
**Analogue Evaluation Cocaine**	,795 (2,32)	**,963 (2,57)**	**,320 (1,31)**	,023*	**-**
**STAI-State**	23,90 (13,77)	23,11 (13,91)	26,30 (13,35)	,157	,157
**STAI-Feature**	28,41 (13,04)	**27,08 (12,82)**	**32,10 (13,02)**	,018*	,036*
**BDI**	1,61 (1,19)	1,31 (1,14)	1,51 (1,12)	,142	–

The mean score between men and women are represented along the s.d (between brackets). When significant differences exist between groups, they were signed in bold Font and (*). FDR, False Discovery Rate was calculated in those dependent variables with subscales.

Independent T test comparing substances groups revealed significant differences in age by Alcohol and Alcohol and Cocaine Use Disorder, t(210)=3,537, p=<,001, 95% C.I. (2,654/9,340). In average, Alcohol Use patients are older (M=49,56, SD=11,27) than Alcohol and Cocaine patients (M=43,56, SD=9,40). *Total BIS-11*, [t(199)=-2,587, p=.005, 95% C.I. (-8,001/-1,080)], as well as *Motor Impulsivity* [t(199)=-2,644, p=,004, 95% C.I. (-3,470/-,505)] and *Unplanned impulsivity* [t(199)=-2,720, p=,004, 95% C.I. (-3,654/-,577)] showed statistically significant differences, even when correction with FDR. Average punctuation tends to be higher on Alcohol and Cocaine compared to Alcohol group (Mean and Standard Deviations can be consulted on **Table 2**).

**Table 2:** Mean scores between Alcohol Use Disorder (AUD) and Alcohol and Cocaine Use Disorder (ACUD) for age, BIS-11 and ASRS tests.

	AUD (n=153)	ACUD (n=51)	*p*	FDR
**AGE**	**49,56 (11,27)**	**43,56 (9,40)**	,010*	**-**
**TOTAL BIS-11**	**68,37 (10,98)**	**72,91 (11,16)**	,005*	,02*
Cognitive Impulsivity	20,42 (3,87)	21,19 (3,65)	,105	,105
Motor Impulsivity	**21,53 (4,88)**	**23,52 (4,25)**	,009*	,012*
Unplanned impulsivity	**26,66 (4,81)**	**28,76 (4,95)**	,007*	,014*
**ASRS**	10,16 (5,96)	11,20 (4,98)	,126	–

The mean score between groups are represented along the s.d (between brackets). When significant differences exist between groups, they were signed in bold Font and (*). FDR: False Discovery Rate was calculated in those dependent variables with subscales.

Comparing the percentage of men (22,3%) and women (34%) scoring above the cutoff point on the ASRS, no significant differences were found, Chi Square(1)=2,911, p=,088. Same analysis comparing Alcohol (21,9%) and Alcohol and Cocaine (32,2%) also did not report significant differences, Chi Square(1)=2,509, p=,113).

The correlation between the ASRS test score and the main scale of the BIS-11, as well as its subscales, shows relation (see **Table 3**) for the total sample. These results maintain a correlation when the analyses are conducted using only women’s group (p between 0.000 and 0.004). In men, the main scale and the *Motor Impulsivity* subscale do not show significant relationships.

**Table 3:** Correlation between the ASRS test about ADHD screening and impulsivity.

	Total			Men			Women		
	R	p	FDR	R	p	FDR	R	p	FDR
**TOTAL BIS-11**	,160	,024*	,024*	,034	,676	,676	**,609**	,000*	,000*
Cognitive impulsivity	,236	,001*	,001*	,151	,066	,088	**,483**	,000*	,000*
Motor impulsivity	,303	,000*	,000*	**,249**	,002*	,02*	**,399**	,004*	,004*
Unplanned impulsivity	,247	,000*	,000*	**,210**	,010*	,008*	**,480**	,000*	,000*

The first part of the table represents the correlation for all the sample and in the second part the sample was divided in Men, Women before we made that correlation. When significant differences exist between groups, they were signed in bold Font and (*). R, R Pearson correlation: unilateral signification. FDR, False Discovery Rate was calculated in those dependent variables with subscales.

When correlating the remaining variables with the ASRS, it was found that in the total study sample, there is a statistically significant relationship between ADHD scores and most of the clinical and personality variables analyzed in this study.

When conducting the analysis by separating men and women, we found several differences between the two groups. Given the number of variables considered, we suggest using **Table 4** as a reference to facilitate clearer interpretation.

**Table 4:** Relation between impulsitiv(Barrat) and other variables, according to gender.

	Total			Men			Women		
	R	p	FDR	R	p	FDR	R	P	FDR
**ZKPQ NEUROTICISM**	,360	,000*	–	,294	,000*	-	,547	,000*	–
**ZKPQ ACTIVITY**	-,181	,010*	,015*	-,140	,089	,133	-,314	,002*	,007*
ZKPQ general activity	-,098	,165	,165	-,059	,478	,478	-,214	,136	,136
ZKPQ working effort	,227	,001*	,003*	-,196	,016*	,048*	-,344	,014*	,021*
**ZKPQ SOCIABILITY**	-,154	,030*	,045*	-,129	,116	,174	**-,280**	,049*	,147
ZKPQ party and friends	-,073	,302	,302	-,043	,602	,602	-,196	,172	,172
ZKPQ isolation intolerance	-1,181	,010*	,030*	-,176	,032*	,096	-,247	,083	,124
**ZKPQ IMPULSIVITY**	,327	,000*	,000*	,369	,000*	,000*	,249	,082	,123
ZKPQ impulsivity	,428	,000*	,000*	,443	,000*	,000*	,434	,002*	,006*
ZKPQ sensations search	,174	,014*	,014*	,222	,006*	,000*	,067	,642	,642
**ZKPQ AGRESIVITY-HOSTILITY**	,338	,000*	–	,318	,000*	–	,393	,005*	–
**ZKPQ INFREQUENCY**	-,142	,045*	–	-,042	**,613**	–	-,437	,002*	–
**Stai-State**	,395	,000*	,000*	,391	,000*	,000*	,426	,002*	,002*
**Stai-Feature**	,484	,000*	,000*	,470	,000*	,000*	,544	,000*	,000*
**Eva: Cocaine**	,184	,009*	–	,230	,005*	–	-,032	,826	–
**Eva: ALCOHOL**	,313	,000*	–	,283	,000*	–	,430	,002*	–
**BDI**	,454	,000*	–	,450	,000*	-	,393	,019*	–

When significant differences exist between groups, they were signed in bold Font and (*). R: R Pearson correlation: unilateral signification. FDR: False Discovery Rate was calculated in those dependent variables with subscales.

## Discussion

Greater Total, Motor and Non-planning Impulsivity were registered (according to BIS-11’s scale) in the group with Alcohol and Cocaine Use Disorder than in the group with Alcohol Use Disorder. We have confirmed that there is a strong association of ADHD with substance misuse and impulsivity. From a gender perspective, ADHD has been found to have greater association for impulsivity among women. In the group of women, we observed that greater impulsivity involved less sociability and participation in activities. Both men and women present a direct association between impulsivity and alcohol craving, as well as with neuroticism, sensation seeking, impulsivity and aggression-hostility; and an inverse association with work energy and infrequency. In the group of men, impulsivity was associated with lower intolerance to isolation and cocaine craving. In the affective sphere, high levels of impulsivity were associated with anxiety (measured with the STAI) and depression criteria (BDI) in both men and women.

The high levels of impulsivity found across the entire sample agree with the literature, because among the general population, motor impulsivity (propensity to react rapidly to a stimulus without considering the consequences) has been associated with drug use (4, 41). There are statistically significant differences regarding total impulsivity between the group of patients with alcohol and cocaine use disorder and those who only had alcohol use disorder. This was also observed in the subscales of motor and non-planning impulsivity. Impulsivity has been associated with the use of drugs (42), and particularly with cocaine (3, 8, 24).

With regard to gender, total impulsivity and its different subtypes were similar in men and women in the population of our study. This is different from what has been observed in the general population, in which men show greater impulsivity (13). In our sample, impulsivity was the same in both groups, and this could be due to the frequency of ADHD and alcohol use, because women have been reported to present more impulsivity, particularly after prolonged alcohol consumption (43).

The association between impulsivity and craving also presents differences regarding gender. Both women and men present a direct association between impulsivity and alcohol craving, but men also present an association with cocaine craving. Preclinical studies have analyzed the differences in alcohol craving between genders (44) and exposure to drugs has been reported to have different effects depending on gender (45). After consumption, women report a stronger feeling of stress and higher levels of craving (46). However, further studies should be conducted on impulsivity and craving according to gender, because this is an essential aspect in the relapse process that may involve a poor evolution (47).

At an affective level, high levels of impulsivity have been associated with anxiety and depression criteria in both men and women. However, gender differences have been reported in the neuroendocrine adaptation to stress and reward systems that may mediate women’s susceptibility to the use of drugs and relapses (48), and to mood and anxiety disorders throughout life, which are significantly higher among women than among men, both with and without substance use disorders (49). The similarities between genders observed in our study could be associated with the severity of the disorders in our sample.

We have confirmed a strong association between ADHD and substance addiction and impulsivity. The highest scores in the ADHD self-report were associated with higher impulsivity scores in both drug use groups, and this confirms previous findings of a high coexistence of alcohol and cocaine use disorders and ADHD (17, 28, 31, 50).

No differences were observed regarding the ASRS and impulsivity between the ACUD and the AUD groups. It had previously been reported that no differences had been found comparing patients who used cocaine, cannabis, or both (51). This suggests that there are factors, other than impulsivity and ADHD, which have an influence on the choice of the main substances of addiction.

With regard to gender, the presence of ADHD in women is directly associated with total impulsivity and all impulsivity subscales. However, in men an association was only found with the subscales of motor and non-planning impulsivity.

Since the scores were similar for both genders, it could be suggested that in women with ADHD impulsivity levels increase more than in men, due to a ceiling effect for men, in which the presence of ADHD barely affects impulsivity, which already presents high levels. This would explain why ADHD has greater explanatory power for impulsivity in women, and why their levels are similar to those observed in men. This reveals the significance of early assessment and detection of this disorder in women. In this sense, significant sex-by-symptom interactions between diagnostic and treatment status for hyperactivity/impulsivity and behavior problems had already been described (52). One possible reason could be that drug use may affect the brain of men and women differently, and this could explain the higher impulsivity in women than in men (53) and the fact that in our sample levels were similar to those observed in men.

Gender differences were also observed in the accompanying psychopathology, because women with ADHD are more likely to present borderline personality disorder, which may account for the drug use. Men, on the other hand, have a greater risk of developing antisocial personality disorder associated with drug use (54). Similarities between genders have been found in ADHD with behavioral disorders, depression, bipolar disorder and schizophrenia, in which men and women have an increased risk of SUD. However, a reduced risk was found for men with autism spectrum disorder (30).

Finally, SUD in ADHD patients has been associated with symptoms of hyperactivity-impulsivity and emotional dysregulation. Self-medication for ADHD via drug use has been put forward as a potential explanation, and early diagnosis and treatment of ADHD have been suggested as a preventive strategy against drug use (31).

Considering the results from the ZKPQ, both genders present a similar direct association between impulsivity and neuroticism, sensation seeking, impulsivity and aggression-hostility, and an inverse association with work energy and infrequency. However, women with higher impulsivity presented lower sociability and activity participation, while in men the inverse association was found with isolation intolerance. Interpreting these differences is complex because there are very few studies that assess the gender differences for personality traits based on the presence of impulsivity. There are studies that have found an association between traits such as neuroticism and aggression-hostility with a greater severity of the addiction and the presence of psychotic symptoms (55, 56), and between impulsivity and ADHD (3). What seems evident is that the presence of impulsivity creates opposite effects: it decreases sociability and activity in women, which is associated to greater isolation, whereas in men it could be associated with lower isolation tolerance. This suggests that different approaches are required for each group.

Even though there is an increasing amount of evidence for the need to create specific adaptations to treatments depending on gender due to structural and neurochemical differences (45, 46, 57), to date, most treatment models for substance use disorder have been designed primarily for men, and they are mainly based on their symptoms and consumption patterns. Consequently, women with ADHD may be more easily missed in the ADHD diagnostic process and treatment unless they have prominent externalizing problems (52). In this sense, being aware of the drug use patterns in women may be useful to detect dual pathologies earlier and to implement specific gender-based interventions aimed at providing adapted information and services that meet their needs in areas such as child rearing, domestic violence, sexual trauma and psychiatric comorbidities.

Our results allow us to draw some clinical implications, because psychoeducation could be used to understand the influence of ADHD and of impulsivity and isolation, as well as the role they play in cocaine and alcohol use. In addition, behavioral techniques could be implemented to approach delay of drug use, distress tolerance and emotion regulation (58). In women, this could improve the capacity to identify internal and external signals that appear before prior to the risk of impulsive behavior. That is, it would make it easier to know the role of impulsivity and sensation-seeking while providing therapeutic tools to manage these impulses in a wider therapeutic context.

With regard to limitations, our study was conducted in only one site, the University city of Salamanca; therefore, the sociodemographic and cultural characteristic of the population under study may not be generalizable to other places. However, women in studies such as this are often underrepresented given that, in clinical settings, the number of women is usually lower (3, 56). Also, we did not employ neurobiological investigations, such as neuroimaging, to establish the links between our findings and brain functions. Furthermore, we did not include patients with comorbid severe mental disorders, such as schizophrenia or bipolar disorder; thus, our work may need to be replicated with other, more severe, clinical groups. However, our study was carried out with a homogeneous clinical sample attending an outpatient clinic; therefore, the results are representative of routine, real-world clinical practice. In fact, our findings emphasize the consequences of having a diagnosis of ADHD for women also suffering from an addiction, and the importance of its early detection and treatment. Studies with focus on the causal mechanisms (and associated gender differences) between suffering ADHD and the development of alcohol and/or cocaine dependence are still warranted.

However, the study was conducted in a homogeneous sample of patients who are alcohol-dependent or alcohol- and cocaine-dependent in an outpatient treatment center, which means that data are representative of routine clinical practice. Consequently, the results are representative of the clinical activity in a real clinical setting. and could help to emphasize the relevance of ADHD in the severity of women suffering addiction and point out the relevance of including the early detection and its treatment in the clinical protocols.

We may conclude that there are differences regarding total impulsivity depending on the substance of addiction, with higher levels of impulsivity in the cocaine and alcohol group. A strong association was observed between ADHD and substance use disorders. Even though no gender differences were found for impulsivity, there are differences regarding the influence of ADHD on impulsivity, which is more relevant among women. ASRS has a clearly higher explanatory power for impulsivity in the group of women who are drug users. In addition, some personality traits and craving seem to present different patterns depending on gender and impulsivity.

The relevance of ADHD in women is key to understand the presence of impulsivity and the complications associated with it, which means that it must be studied and explored systematically. In the future, follow-up studies should be conducted that include the relevance of and relationships between ADHD and gender and relapses among AUD and CUD in patients seeking treatment.

## Data Availability

The raw data supporting the conclusions of this article will be made available by the authors, without undue reservation.
